# Fructose Metabolism in Cancer: Molecular Mechanisms and Therapeutic Implications

**DOI:** 10.7150/ijms.108549

**Published:** 2025-06-09

**Authors:** Xinyi Chen, Mu Yang, Lu Wang, Jingyao Tu, Xianglin Yuan

**Affiliations:** Department of Oncology, Tongji Hospital, Tongji Medical College, Huazhong University of Science and Technology, Wuhan, Hubei, China.

**Keywords:** fructose metabolism, tumor metabolism, metabolic reprogramming, ketohexokinase, aldolase, glucose transporter

## Abstract

Metabolic reprogramming enables cancer cells to adapt to the tumor microenvironment, facilitating their survival, proliferation, and resistance to therapy. While glucose has long been considered the primary substrate for cancer cell metabolism, recent studies have highlighted the role of fructose as an alternative carbon source. Fructose metabolism, particularly through key enzymes such as ketohexokinase (KHK) and aldolase B (ALDOB), along with the fructose transporter GLUT5, supports tumor growth, metastasis, and therapeutic resistance. This review explores the mechanisms by which fructose metabolism influences cancer progression, focusing on its metabolic pathways and its impact on the tumor microenvironment. By promoting glycolysis, lipid biosynthesis, and nucleotide production, fructose metabolism enhances the metabolic adaptability of cancer cells, especially in glucose-deprived conditions. A comprehensive understanding of these processes offers potential insights into therapeutic strategies targeting fructose metabolism for cancer treatment. However, further studies are required to fully elucidate the complex role of fructose in various malignancies.

## 1. Introduction

Through metabolic reprogramming, cancer cells can progressively acquire necessary adaptations for survival within the tumor microenvironment (TME). These adaptations are vital for supporting elevated synthetic demands and aggressive proliferation, consequently enhancing resistance to anti-tumor immunity. Otto Warburg's investigations into cancer cell metabolism in the 1920s identified an unexpected phenomenon: cancer cells were found to convert glucose to lactate more effectively than normal tissues, even in well-oxygenated environments. Warburg theorized that this increased glycolysis was due to underlying mitochondrial dysfunction, a concept now known as the Warburg effect 1, 2. Glycolysis, although less efficient in ATP generation than mitochondrial oxidative respiration, is essential for generating intermediates utilized for synthesizing amino acids, lipids, and nucleotides. These intermediates, therefore, support the increased demands of tumor cells for proliferation and enduring therapeutic resistance 3. Investigating the interplay between cancer glycolysis and the metabolic dysregulation characteristic of metabolic syndrome could potentially identify novel therapeutic targets and deepen the understanding of their interrelationship.

Glucose, a fundamental substrate for glycolysis in cancer cells, provides energy, supports the synthesis of metabolites such as serine, aspartate, nucleotides, and fatty acids, and contributes to redox regulation 4-6. In tumor cells, rapid glucose consumption often leads to its conversion to fructose via the polyol pathway, generating an endogenous fructose supply that supports metabolism in stress conditions​ 7. However, emerging evidence indicates that oxygen deprivation (hypoxia), rather than glucose scarcity, is the primary trigger for this endogenous fructose production 8. The TME is frequently hypoxic, which independently activates the polyol pathway and drives the conversion of glucose to fructose even when glucose is abundant​ 9. Under hypoxia, hypoxia-inducible factor-1α (HIF-1α) upregulates aldose reductase (AR) and sorbitol dehydrogenase (SDH), the key enzymes converting glucose to sorbitol and then to fructose 10, 11. Consequently, hypoxic tumor cells accumulate high levels of sorbitol and fructose; for example, chronic hypoxia caused an >80-fold increase in intracellular fructose (with ~3-fold rise in sorbitol) in glioblastoma cells 12. Notably, this de novo fructose synthesis occurs even with ample glucose, showing that oxygen deprivation alone can redirect glucose into fructose as an adaptive mechanism. Consistently, hypoxia-induced AR/SDH activity is cytoprotective: AR upregulation under hypoxia helps prevent cell death, whereas AR inhibition reduces HIF-1α accumulation and impairs survival signaling 10, 13. Fructose produced via this HIF-1α-driven polyol pathway sustains glycolytic energy output under low oxygen, maintaining cancer cell viability and proliferation despite impaired oxidative phosphorylation 12, 14. Indeed, fructose supplementation mitigates hypoxia-induced necroptosis in colorectal cancer cells by boosting glycolytic intermediates and ATP, highlighting fructose's role in hypoxic metabolic adaptation 9​^15^. In summary, a hypoxia-driven fructose metabolic program, orchestrated by HIF-1α, allows tumors to endure oxygen deprivation by funneling glucose into fructose metabolism.

To compensate for this energy deficiency, cancer cells commonly employ both dietary and endogenously generated fructose-1-phosphate as an alternative energy and carbon source to sustain the glycolytic process and support the synthesis of biomacromolecules. It has been established by recent studies that tumor cells have the capability to metabolize fructose directly as an alternative carbon source 14, 16. Fructose, a freely interchangeable monosaccharide with glucose, is commonly found in human diets. Since the introduction of high-fructose corn syrup (HFCS) into the food processing industry in the 1970s, there has been a significant increase in fructose consumption 17. Over the past two centuries, the rise in dietary fructose intake has been closely associated with the development of diabetes, obesity, and hepatic steatosis 18. Notably, extensive epidemiological studies and animal experiments have demonstrated that high fructose intake is linked not only to metabolic syndromes such as obesity and non-alcoholic fatty liver disease but also closely related to the incidence and progression of various cancers 19. Excessive intake of fructose has been shown to promote the development and malignancy of various types of tumors, thereby adversely affecting the prognosis of cancer patients 20-22. Apart from recognized metabolic pathways, dietary fructose is rapidly absorbed in the intestines and subsequently concentrated and metabolized in the liver 23, 24. High fructose intake can exacerbate intestinal barrier dysfunction, potentially leading to endotoxemia and persistent inflammatory stimuli, ultimately resulting in liver cancer 24. Moreover, even in the absence of obesity and metabolic syndrome, HFCS can initiate glycolysis and enhance fatty acid synthesis, facilitating the development and progression of intestinal tumors 20. Therefore, a more thorough understanding of the mechanisms by which fructose promotes the malignant progression of cancer could provide critical insights for more effective cancer prevention and treatment.

The review evaluates the mechanisms through which fructose metabolism affects cancer progression and metastasis. By investigating the metabolic pathways and critical enzymes, along with the impact of fructose on the tumor microenvironment and therapy resistance, this review aims to comprehensively elucidate fructose's role in cancer and its potential for preventive and therapeutic applications. Insights into fructose's contribution to cancer cell metabolism emphasize its role in promoting cell survival, particularly via pathways that involve ketohexokinase (KHK) and aldolase B (ALDOB). Additionally, the upregulation of specific transport proteins such as glucose transporter 5 (GLUT5) and glucose transporter 8 (GLUT8) enhances the metabolic adaptability of cancer cells. Understanding fructose metabolism is critical to developing treatments that potentially suppress tumor progression and improve survival outcomes for patients. Such knowledge is fundamental to the progress in cancer therapies and prevention.

## 2. Fructose Metabolic Pathways and Their Role in Tumor Cells

### 2.1. Fructose Catabolism: Key Enzymes and Metabolic Products

Under physiological conditions, fructose catabolism (“fructolysis”) shares many enzymes with glycolysis, but in certain tissues and contexts it proceeds via distinct routes. Hypoxia in tumors strongly induces fructose production through the polyol pathway, as discussed above. HIF-1α activation elevates AR and SDH, leading to an influx of sorbitol and fructose in hypoxic cancer cells 14, 25. This hypoxia-driven polyol flux occurs even without glucose deprivation and provides an alternate fuel to maintain glycolysis when oxygen is limited 26. Interestingly, if HIF-1α is inhibited, hypoxic cancer cells engage compensatory pathways (Myc, PI3K-Akt, AMPK) to sustain glycolysis and fructose utilization​ 27. Thus, while HIF-1α is a central regulator of fructose metabolism in hypoxia, tumor cells can adapt to ensure fructose catabolism (and survival) even when HIF signaling is compromised 28​. Overall, HIF-1α-mediated fructose production supports glycolytic ATP generation under low oxygen, promoting cancer cell viability despite impaired oxidative phosphorylation 29. Inhibiting this adaptation (e.g. via AR blockade) can diminish HIF-1α levels and tumor cell survival under hypoxia​, underscoring the polyol pathway's importance in hypoxic tumor metabolism 13.

Fructose, a monosaccharide, is predominantly transported into cells via GLUT5, a high-affinity fructose transporter, encoded by the solute carrier family 2 member 5 (SLC2A5) gene, independent of insulin stimulation 30. Fructose metabolism is primarily confined to certain tissues including the liver, adipose tissue, and small intestine 31, where heightened expression of the specialized fructose transporter 5 (GLUT5) correlates strongly with the malignant progression of tumors and adverse clinical prognoses 32-34. Recent studies have demonstrated that in the context of glucose scarcity within the tumor microenvironment, fructose may function as an alternative energy source, compensating for and potentially facilitating tumor development and metastasis. For instance, under glucose-limited conditions, it was observed that acute myeloid leukemia (AML) cells 35, pancreatic cancer cells 36, and lung cancer cells 34 augment fructose metabolism through enhanced expression and activity of the fructose transporter GLUT5, supporting malignant proliferation. Fructose can also facilitate the metastasis of colon cancer to the liver through the KHK-ALDOB pathway 37. Ketohexokinase-A (KHK-A), functioning as a nuclear protein kinase, facilitates fructose-induced metastasis in breast cancer 38. Intracellularly, fructose is phosphorylated by KHK to generate fructose 1-phosphate (F1P), utilizing adenosine triphosphate (ATP) as the phosphate donor 39. ALDOB subsequently cleaves F1P into dihydroxyacetone phosphate (DHAP) and glyceraldehyde (GA), after which triose kinase metabolizes these intermediates into glyceraldehyde 3-phosphate (GAP), advancing further into glycolysis. Thus, fructose metabolism circumvents the initial regulatory steps of glycolysis, leading to enhanced lipid production compared to the synthesis achieved through glycolysis 40.

Fructolysis, diverging from glycolysis, bypasses phosphofructokinase-1 (PFK-1), an enzyme essential for catalyzing the transformation of fructose 6-phosphate (F6P) into fructose 1,6-bisphosphate, a critical rate-limiting step in glycolysis. Fructose 2,6-bisphosphate, derived from F6P by PFK-2, activates PFK-1 41. Feedback inhibition of phosphofructokinase-1 occurs in response to increased levels of ATP and citrate, alongside reductions in pH and oxygen availability 42. Therefore, the rate of fructolysis substantially exceeds that of glycolysis. Under specific conditions, including in certain cancer cells, hexokinase (HK) can phosphorylate fructose to fructose 6-phosphate (F6P), thus enabling its participation in the glycolytic pathway 43. Recent studies have indicated that fructose exerts a more adverse effect concerning health status than glucose. This difference in impact may be attributed to their distinct chemical structures and metabolic pathways. Fructose, containing a ketone group at the second carbon, contrasts with glucose, characterized by an aldehyde group on the first carbon. Fructose metabolism, unlike that of glucose, which is insulin-regulated, occurs predominantly through KHK in an insulin-independent manner 44. Furthermore, KHK, lacking feedback inhibition, rapidly metabolizes fructose to F1P, an intermediate known for its potential toxicity when accumulated.

### 2.2. Comparison of Fructose and Glucose Metabolism

**Figure 1:**
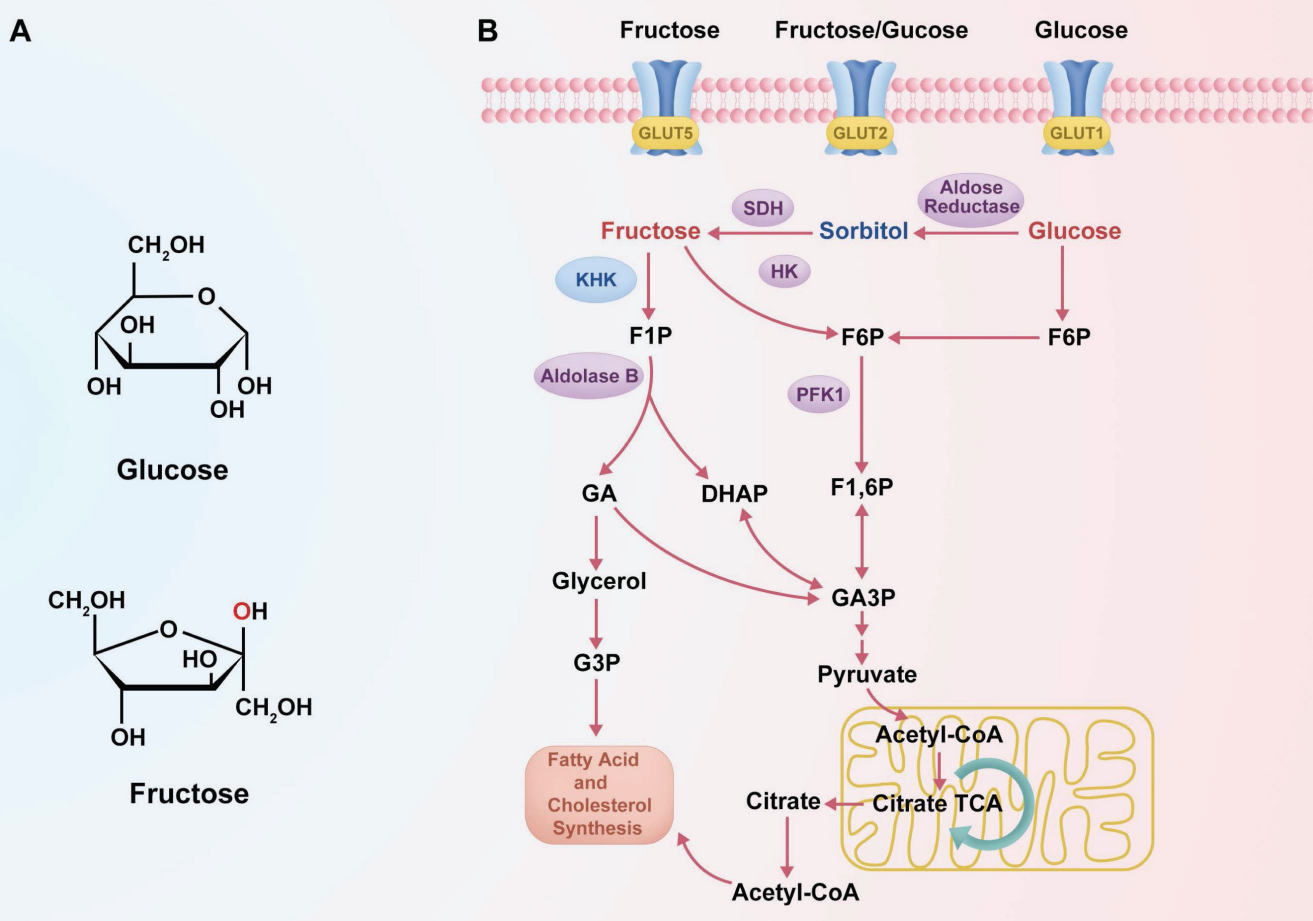
** Metabolic pathways of fructose and glucose. (A)** Structural differences between glucose and fructose, with the hydroxyl group on the second carbon highlighted in red. **(B)** Schematic diagram showing the distinct transporters and metabolic pathways of glucose and fructose. Fructose enters cells via GLUT5 and is phosphorylated by KHK to form F1P, bypassing PFK1 regulation. Glucose is transported via GLUT1/GLUT2 and converted to F6P, entering the classical glycolytic pathway.

Despite both being hexose monosaccharides (C₆H₁₂O₆), fructose and glucose exhibit distinct structural configurations and enter metabolic pathways via different enzymatic mechanisms 23. These differences in molecular structure and metabolic entry points are summarized in **Figure 1**, which schematically illustrates the distinct transporters, phosphorylation enzymes, and downstream metabolic pathways for glucose and fructose. Fructose is a ketohexose with a ketone group at the second carbon, whereas glucose is an aldohexose with an aldehyde group at the first carbon, and this structural variation necessitates differential enzymatic handling during metabolism 23. Glucose metabolism is tightly regulated by insulin and begins with phosphorylation by hexokinase, proceeding through the glycolytic pathway where PFK-1 serves as a key rate-limiting checkpoint 45. In contrast, fructose metabolism—termed “fructolysis”—is largely insulin-independent and is initiated primarily by KHK, especially the KHK-C isoform abundantly expressed in hepatocytes 46, 47. KHK rapidly phosphorylates fructose into F1P, bypassing the regulatory checkpoint at PFK-1, and, unlike hexokinase, its activity is not inhibited by intracellular ATP, citrate, or acidic pH 30. As a result, fructose bypasses the key regulatory node in glycolysis, enabling a sustained and unregulated influx of carbon into glycolytic and lipogenic pathways, even under conditions that suppress glucose metabolism 30, 46, 48, 49. Indeed, several studies have demonstrated that fructose catabolism yields glycolytic intermediates and lipid precursors at a higher rate than equimolar glucose, particularly under specific cellular conditions 50. For example, in hepatocytes, fructose administration has been shown to enhance de novo lipogenesis more significantly than glucose, a phenomenon attributed to its unregulated metabolic entry downstream of PFK-1 51. These observations underscore that, although fructose and glucose share an identical molecular formula, their metabolic regulation, enzymatic handling, and downstream effects differ profoundly.

Fructose and glucose also differ in their primary sites of metabolism and transport mechanisms within the body. Glucose serves as a universal energy source, widely distributed to tissues such as muscle and brain, and can be stored in the liver as glycogen or converted into fructose via the polyol pathway under specific conditions 31. In contrast, fructose is primarily metabolized in the liver. After ingestion, dietary fructose is absorbed in the small intestine by facilitative transporters, mainly GLUT5, with additional contribution from GLUT2, and is then efficiently transported into hepatocytes via GLUT2 52, 53. This hepatic first-pass metabolism enables rapid conversion of fructose to F1P by KHK upon its entry into liver cells 32, 39. The rapid phosphorylation of fructose maintains a concentration gradient that facilitates continued absorption from the portal circulation into hepatocytes 14, 54. In comparison, glucose absorption occurs through both sodium-dependent (e.g., SGLT1) and facilitative transporters (e.g., GLUT1, GLUT4), and is tightly linked to insulin secretion and action 55. Fructose, however, elicits only minimal insulin response, a distinction with important systemic implications. Because insulin and leptin—hormones critical for regulating appetite and energy balance—are only modestly influenced by fructose intake, high fructose consumption may fail to activate satiety pathways 56, 57. This can result in increased caloric intake and adiposity, both of which are known contributors to cancer risk. Thus, while glucose is utilized by virtually all tissues and is subject to hormonal feedback regulation, fructose is preferentially taken up and metabolized in the liver under relatively minimal endocrine control. These metabolic distinctions in absorption and regulatory signaling (see Figure 1A) support the notion that fructose may provide tumor cells with a metabolic advantage not observed with glucose. As further illustrated in Figure 1B, the distinct metabolic routing of fructose—bypassing key glycolytic checkpoints—facilitates sustained carbon influx into anabolic pathways, particularly under hypoxic or energy-restricted conditions, which may be critical for tumor progression. In this context, the differences between fructose and glucose metabolism become particularly significant in cancer, where tumor tissues often exist in a hypoxic and acidic microenvironment, which alters metabolic preferences. Under low oxygen conditions, cancer cells upregulate the polyol pathway as an adaptive response—driven by HIF-1α-mediated activation of aldose reductase and sorbitol dehydrogenase 13. This pathway converts abundant intracellular glucose into fructose, and subsequently into F1P, even when glucose is not limiting 58, 59. Thus, it is hypoxia—not glucose deprivation—that primarily stimulates this metabolic shift, enabling tumor cells to redirect glucose into fructose production as an alternative carbon source. The resulting endogenous fructose pool ensures a steady supply of F1P within cancer cells 14, 60. This preferential routing of glucose to fructose under hypoxic stress is part of a broader HIF-1α-driven metabolic rewiring program that enhances glycolytic flux, suppresses mitochondrial oxidation, and promotes lactate production, thereby ensuring sustained ATP generation even under oxygen-limited conditions. These adaptations are crucial for tumor cell survival, proliferation, and chemoresistance in the hypoxic tumor microenvironment 61. Because fructose metabolism proceeds independently of insulin, tumor cells can continue generating F1P under stress conditions such as insulin resistance or impaired glucose uptake. More importantly, F1P enters glycolysis downstream of PFK-1, effectively bypassing this critical rate-limiting checkpoint 36, 37. Under normal conditions, PFK-1 activity is allosterically inhibited by high levels of ATP, citrate, or low pH, leading to reduced glycolytic throughput 30​. These inhibitory signals are frequently encountered in hypoxic tumors, where acidosis and metabolic reprogramming are common features 62. Fructose metabolism circumvents these constraints. Once phosphorylated by KHK, F1P proceeds through glycolysis unhindered by energy status feedback, allowing carbon flux to continue even under metabolic suppression 31, 58. This bypass is mechanistically underpinned by the ability of fructose to avoid PFK-1 regulation, enabling a rapid and unregulated glycolytic influx. Notably, fructose metabolism via KHK causes acute ATP depletion and phosphate loss, which activates downstream lipogenic pathways and contributes to a pro-anabolic metabolic environment, particularly under conditions of energy stress 63. This capacity confers a significant advantage to cancer cells, enabling them to sustain ATP production, preserve glycolytic intermediates, and fuel anabolic pathways 14, 64. In hypoxic or acidic environments, cancer cells metabolizing fructose can maintain biosynthesis of nucleotides, amino acids, and lipids even as glucose-driven glycolysis is downregulated 42, 64. By bypassing PFK-1, fructose-driven glycolysis remains active when the Warburg effect would otherwise be attenuated, allowing tumors to continue lactate and energy production under cellular stress 35, 36. Notably, recent studies have confirmed that fructose metabolism accelerates glycolytic flux in hypoxia, thereby supporting cancer cell viability and proliferation under oxygen or pH limitations 14, 42, 64. This unique regulatory bypass underscores why fructose acts as a "metabolic shortcut", enhancing tumor survival by circumventing the key bottleneck of PFK-1 65, 66. This phenomenon reflects an evolutionarily selected glycolytic phenotype, in which tumor cells consistently upregulate glycolysis—even under normoxia—not because of metabolic inefficiency, but because it provides a survival advantage in hypoxic and acidic microenvironments through acid-mediated selection and enhanced invasiveness 67.

Beyond these regulatory considerations, fructose and glucose diverge in how they channel carbon into downstream biosynthetic programs in tumor cells 36. A key distinction lies in their differential routing through the pentose phosphate pathway (PPP), a central metabolic hub for nucleotide and redox metabolism. Glucose primarily engages the oxidative branch of the PPP via glucose-6-phosphate dehydrogenase (G6PD), generating NADPH to support antioxidant defense and lipid biosynthesis 68. Fructose, by contrast, more efficiently feeds the non-oxidative branch, enhancing flux through transketolase (TKT) to generate ribose-5-phosphate and uric acid, thereby promoting nucleotide synthesis 36. This difference has functional consequences in tumors. In pancreatic cancer cells, both glucose and fructose support proliferation, but fructose selectively upregulates non-oxidative PPP activity, leading to increased ribose and uric acid production, while glucose preferentially elevates lactate and CO₂ output via glycolysis and oxidative PPP flux 36. Liu *et al.* demonstrated that fructose-grown pancreatic tumor cells exhibited a marked increase in TKT-mediated flux, compared to glucose-grown cells which primarily activate G6PD in the oxidative arm 36. This preferential use of fructose for nucleotide synthesis may confer a proliferative advantage in rapidly dividing cells. A similar pattern is observed in lung adenocarcinoma. Weng *et al.* reported that fructose more effectively supports ATP generation and fatty acid synthesis than glucose in these cells, correlating with enhanced lipid accumulation and proliferation 33, 34. These findings suggest that fructose not only circumvents key regulatory checkpoints but also optimally supplies carbon for anabolic processes critical to tumor growth. Such metabolic specialization may help explain how tumors in distinct tissues exploit different sugar substrates. While glucose remains the dominant energy source in many cell types, tumors exhibiting elevated expression of fructose transporters such as GLUT5 (SLC2A5) may preferentially utilize fructose, particularly under nutrient stress or hypoxia 33, 53. Taken together, these findings support the notion that fructose serves as a distinct metabolic substrate, particularly effective in fueling biosynthesis and energy metabolism in certain cancers.

Fructose may also promote more aggressive cancer phenotypes compared to glucose. In breast cancer cells (MDA-MB-468), fructose as a metabolic substrate induces a more aggressive phenotype, significantly enhancing cellular adhesion and migration, whereas glucose does not elicit comparable pro-metastatic behavior 69. These functional differences suggest that fructose metabolism may facilitate enhanced invasive potential in certain tumor types. Consistently, in vivo studies have demonstrated that high-fructose diets accelerate tumor progression. For example, in a murine model of breast cancer, a sucrose-rich diet (high in fructose content) led to significantly larger primary tumors and a higher incidence of lung metastases compared to isocaloric diets in which glucose or starch was the primary carbohydrate source 69, 70. These findings implicate dietary fructose as a driver of both tumor proliferation and metastatic dissemination 20, 70. Fructose and glucose also exert divergent effects on systemic metabolism, which may indirectly influence cancer progression. Fructose has been shown to exacerbate insulin resistance and hepatic steatosis to a greater extent than glucose 57, 59. In a comparative study, Softic *et al.* reported that in the context of a high-fat diet, fructose supplementation markedly increased hepatic lipogenesis and impaired fatty acid oxidation, resulting in insulin resistance. In contrast, glucose supplementation led to increased liver triglyceride accumulation without causing insulin resistance 59, 71. Additional experimental evidence supports fructose-specific metabolic toxicity. In rodent models, inhibition of KHK—responsible for F1P generation—was shown to ameliorate fructose-induced hepatic dysfunction, underscoring the enzyme's central role in mediating fructose-related metabolic stress 59, 72, 73. Because obesity, insulin resistance, and the metabolic syndrome are well-established risk factors for various malignancies, the distinct systemic effects of fructose compared to glucose may contribute to cancer initiation or progression 51. Importantly, fructose does not elicit a significant postprandial insulin or leptin response, both of which are critical hormonal regulators of appetite and energy balance 56, 74. As a result, chronic fructose consumption may fail to trigger satiety signals, promoting excess caloric intake, adiposity, and hormonal dysregulation. Over time, such effects can contribute to a pro-tumorigenic internal environment characterized by hyperinsulinemia, systemic inflammation, and metabolic stress 75, 76.

In summary, while glucose remains the primary metabolic fuel for most somatic cells, fructose provides cancer cells with a unique metabolic advantage due to its insulin-independent entry and distinct enzymatic processing. Fructose metabolism proceeds through F1P, which enters glycolysis downstream of PFK-1—a key regulatory checkpoint allosterically inhibited by high ATP, citrate, and acidic pH 30, 77. These inhibitory conditions are commonly present in hypoxic tumor microenvironments, where elevated lactate, low pH, and fluctuating energy status attenuate glucose-driven glycolysis 62. By bypassing PFK-1, fructose-derived F1P sustains glycolytic flux even when glucose metabolism is suppressed, enabling continuous ATP production and anabolic support 36, 42, 78. Fructose-driven glycolysis not only supports energy metabolism but also promotes biosynthetic pathways through enhanced availability of triose phosphates and intermediates feeding into the non-oxidative PPP, especially via transketolase activation 36, 79. This promotes ribose synthesis and nucleotide production, which are essential for proliferation in rapidly dividing cells 36. In this context, fructose has been shown to enhance transketolase activity and nucleotide biosynthesis more effectively than glucose, particularly under low-oxygen or acidic stress conditions 36, 42, 80. Importantly, cancer cells can derive fructose not only from the diet but also endogenously via the polyol pathway, especially under hypoxia-induced HIF-1α activation 13, 58, 60. This internal fructose supply ensures continued metabolic support even in glucose-restricted environments. Moreover, the transport of fructose via GLUT5 and its metabolism through KHK in hepatocytes and cancer cells is not subject to classical endocrine regulation, such as insulin or leptin feedback 46, 54, 56. This allows fructose to escape systemic metabolic control, thereby favoring unregulated growth and proliferation. Beyond intracellular advantages, fructose also exerts systemic effects that may indirectly promote tumor progression. Unlike glucose, fructose fails to elicit a robust insulin or leptin response, which disrupts satiety signaling and can promote increased caloric intake and adiposity 57, 74. Chronic high-fructose consumption has been linked to insulin resistance, hepatic steatosis, and hyperinsulinemia—factors associated with elevated cancer risk 59, 71, 72. Notably, inhibition of KHK has been shown to ameliorate fructose-induced metabolic dysfunction in preclinical models, underscoring its central role in metabolic stress and tumorigenesis 72, 73. Collectively, these findings highlight fructose as a distinct metabolic substrate that confers multiple layers of advantage to cancer cells: bypass of regulatory checkpoints, resistance to metabolic stress, facilitation of anabolic biosynthesis, and insulation from endocrine feedback. Understanding these distinctions is essential for evaluating fructose as a potential metabolic target in cancer therapy and underscores the importance of considering fructose metabolism in both dietary exposure and tumor biology.

### 2.3. Fructose Metabolism and Cancer Cell Metabolic Reprogramming

Studies conducted by the National Institutes of Health (NIH) and the American Association of Retired Persons (NIH-AARP) have indicated limited associations between added fructose and overall cancer risk or cancer-related mortality across major cancer types 81, 82. However, the role of fructose metabolism in cancer cells is multifaceted, with potential mechanisms that may directly influence cancer cell proliferation. Fructose metabolism can modulate tumor cell metabolism, increase reactive oxygen species (ROS), induce DNA damage, and trigger inflammation, all of which contribute to tumor growth 83. Fructose has been linked to an increased risk for specific cancers and may accelerate tumor growth by promoting metabolic reprogramming, unveiling potential oncogenic mechanisms 47. The excessive intake of fructose has been directly related to an increase in various diseases, necessitating a reevaluation of its potential impact on cancer progression in dietary contexts 19. Research by Hui *et al.* indicated that serum fructose levels in pancreatic cancer patients are significantly higher than in healthy individuals, suggesting that fructose may facilitate tumor progression 84. Moreover, studies have found a significant association between increased fructose intake and a higher risk of pancreatic cancer. Fructose may exert its oncogenic effects by promoting insulin resistance and enhancing tumor cell metabolic reprogramming. A daily increase of 25 grams of fructose intake has been linked to a higher risk of pancreatic cancer 85. Additionally, fructose can bypass glucose metabolic pathways, accelerating glycolysis and nucleotide synthesis, thereby further stimulating the growth and proliferation of tumor cells. Fructose may also contribute to tumor progression by increasing uric acid levels, which induce pro-inflammatory responses 86. While fructose supplementation is not a direct cause of liver tumors, it potentially increases the risk of liver cancer by influencing associated metabolic and gene expression pathways 87. Fructose can also support cancer cells in maintaining energy supply under glucose deficiency by activating specific transcription factors and inducing key metabolic proteins. For instance, glioblastoma multiforme (GBM) cells shifted from glycolysis to fructolysis under glucose deprivation, activating transcription factor ATF4 and inducing the expression of fructose metabolic proteins GLUT5 and ALDOB 88. Huang *et al.* found that fructose enhances the glycolytic pathway under hypoxic conditions, inhibiting RIP-dependent necroptosis in colorectal cancer (CRC) cells, thereby promoting tumor cell survival 9. Fructose enhances the expression of ALDOB by activating ChREBP and phosphorylating FoxO1/3α, potentially promoting vascular remodeling and tumor progression 89. Jiang *et al.* discovered that abnormal fructose metabolism following SARS-CoV-2 infection is associated with poor prognosis in CRC patients. Fructose, as an energy source, drives tumor progression 90. Additionally, a high-fructose diet has been shown to alter the intestinal microbiome, which correlates with the development of esophageal adenocarcinoma, suggesting that fructose may accelerate tumor development by adjusting the metabolism and inflammatory responses of the gut microbiome and the host 91. Raman spectroscopy conducted by Kopec *et al.* revealed that fructose supplementation enhanced the metabolic activity of lipid droplets in normal bronchial epithelial cells (BEpiC) and lung cancer cells (A549), suggesting that fructose could facilitate lipid accumulation in cells, possibly accelerating energy storage and growth in tumor cells 92. Fructose also activates signaling pathways in endothelial cells and increases the expression of VEGF in tumor cells, promoting tumor angiogenesis and progression 93. Wang *et al.* demonstrated that a high-fructose diet promotes metabolic dysfunction-associated steatohepatitis (MASH) and its progression to hepatocellular carcinoma (HCC) by inducing gut microbiota dysbiosis 94. Analysis of the effects of acetic acid produced by intestinal microbes on hepatocytes by Esquea *et al.* found that a high-fructose diet promotes the progression of HCC. This mechanism suggests that fructose accelerates liver cancer development by increasing the production of acetic acid by gut microbes, thereby enhancing O-GlcNAcylation in hepatocytes 95. Yuan *et al.* confirmed that long-term fructose intake is associated with increased risk of proximal colon cancer; fructose may exacerbate tumor development by promoting inflammatory and cell proliferation mechanisms 96. In pancreatic cancer cells, fructose supported tumor growth by activating the AMPK-mTORC1 pathway and inhibiting autophagy-related cell death, mediated by the fructose-specific transporter GLUT5, demonstrating the critical role of fructose metabolism in tumor environmental adaptation 97. In prostate cancer cells, the significant upregulation of fructose transport proteins Glut5 and Glut9 enhances the proliferation and invasiveness of cancer cells by promoting the functional expression and transport of fructose, which plays a key role in the progression of prostate cancer 21. This process is further exacerbated when prostate cancer invades the seminal vesicles (SVI), a condition that not only increases the availability of fructose from seminal fluid but also indicates a more aggressive cancer phenotype 98. Carreño *et al.* explored the metabolic pathways of fructose in prostate cancer cells, noting that despite low expression of Glut-1, these cells efficiently utilize fructose to support proliferation and growth, with fructose metabolism potentially promoting cancer cell proliferation through de novo lipogenesis pathways 99. In HCC, tumor endothelial cells enhanced fructose metabolism by upregulating SLC2A5 and KHK, thereby activating the AMPK signaling pathway and mitochondrial function, enhancing endothelial cell function, and exacerbating tumor angiogenesis, growth, and metastasis 79. Research by Zhou *et al.* demonstrated that a high-fructose diet enhances hepatic protein O-GlcNAcylation by elevating levels of glutamate and UDP-N-acetylglucosamine (UDP-GlcNAc) via acetic acid produced by intestinal microbes, thereby promoting the progression of HCC 100. Additionally, Syamprasad *et al.* found that fructose upregulates AKR1B1, facilitating metabolic reprogramming and progression in liver cancer, underscoring the pivotal role of AKR1B1 in tumor development influenced by fructose 101. Hsieh *et al.* also supported this perspective, noting that fructose promotes metastasis in pancreatic cancer by upregulating β-galactoside α2,6-sialyltransferase 1 (ST6Gal1). Fructose not only increased the invasiveness of pancreatic cancer in animal models but also led to the expression of biomarkers associated with poor prognosis in clinical samples 102. Sohn *et al.* discovered that fructose promotes the expression of key self-renewal markers in ovarian cancer stem cells via chaperone-mediated autophagy (CMA). Specifically, fructose increased the activity of LAMP2A and TFEB, changes that are associated with altered expression of genes involved in the ferroptosis pathway, potentially enhancing the malignant characteristics of ovarian cancer stem cells 22. In breast cancer, Fan *et al.* demonstrated that fructose enhances the proliferation and migration of cancer cells, particularly under conditions of glucose deficiency, and a high-fructose diet also increased the risk of metastasis in breast cancer 66. Research by Kuehm *et al.* indicated that fructose increases the expression of HO-1 in melanoma cells, promoting resistance to immunotherapy and aiding the cells in evading immune destruction 103. Taylor *et al.* revealed that fructose plays a significant role in increasing intestinal villi length and enhancing the survival of intestinal cells. This effect, mediated by the regulation of specific metabolic enzymes, enhances nutrient absorption within the tumor microenvironment, highlighting the potential negative impacts of a high-fructose diet on tumor growth 104. In AML, fructose facilitates tumor cell survival in glucose-deprived environments through direct metabolism, leading to the generation of pyruvate and lactate that enter the glycolysis pathway. This enhances tumor cells' metabolic adaptability and viability under specific conditions, suggesting that fructose may propel tumor persistence and progression 105. Additionally, Jeong *et al.* demonstrated that fructose enhanced the survival capabilities of leukemia cells in glucose-deficient conditions by promoting the de novo serine synthesis pathway; inhibition of this pathway could significantly decelerate leukemia progression 43. Hargett *et al.* revealed that fructose promoted liver tumor growth by increasing systemic bile acids, highlighting that modulation of bile acid levels could be an effective strategy to curb fructose-associated liver tumors 106. Furthermore, Nishiguchi *et al.* observed that a high-fructose diet exacerbated colitis symptoms in mice and elevated CRC risk, underscoring the oncogenic potential of fructose in the progression of CRC 107. Such a diet also raised liver cancer incidence in mice lacking the macrophage apoptosis inhibitor (AIM), linking high fructose intake to the development of HCC 108. Assante *et al.* suggested that a high-fructose diet could promote liver cancer risk through epigenetic mechanisms by enhancing hepatic protein acetylation 109. Moreover, Softic *et al.* reported that fructose increased acetylation of mitochondrial proteins in the liver, reducing fatty acid oxidation and leading to impaired liver function and increased tumor risk 110. The impact of fructose on breast cancer is significant as well, with Jiang *et al.* indicating that fructose promotes the 12-LOX pathway and the production of 12-HETE, enhancing the risk and metastasis of breast cancer 70. In CRC, it has been shown that fructose, via the action of KHK-A, promotes liver metastasis of tumors. KHK-A, by phosphorylating PKM2 and inhibiting its activity, enhances tumor cell migration and anti-apoptotic capabilities, highlighting fructose's role in promoting cancer progression 111. In neuroblastoma, fructose has been found to negate the anti-tumor effects of plantain grass extract by maintaining mitochondrial function in N2a cells, particularly through oxidative phosphorylation and mitochondrial membrane potential maintenance. This action potentially contributes to a detrimental effect on tumor survival during neuroblastoma treatment 112. Conversely, Hu *et al.* confirmed that fructose-coated silver microparticles (F-AgÅPs), by inhibiting PDK, alter the glucose metabolism of osteosarcoma cells, promote ROS production, induce apoptosis, and effectively inhibit tumor growth 113. Furthermore, the use of fructose and biotin-conjugated dual-targeted liposomes has shown higher targeting and uptake efficiency in breast cancer cells. These liposomes, through the dual recognition mechanism of GLUT5 and SMVT, facilitate energy-dependent endocytosis, leading to significant drug accumulation within tumor cells 114. Additional studies have shown that the absence of ChREBP affects the expression of intestinal GLUT5, reducing fructose absorption and metabolism, and increasing the concentration of unmetabolized fructose in the gut, which may enhance the survival and proliferation of cancer cells, further emphasizing the significant role fructose may play in cancer progression 115. While the majority of evidence suggests pro-tumorigenic effects of fructose, several studies have demonstrated that fructose can exert anti-tumor effects under specific conditions. For instance, fructose regulates adipocyte metabolism through the mTORC1-dependent pathway, activating leptin production, thereby enhancing the antitumor effects of CD8+ T cells and controlling tumor growth. Studies have shown that in lung cancer patients, high plasma leptin levels associated with fructose concentration improve the antitumor response of CD8+ T cells, revealing the potential application of the fructose-leptin axis in cancer therapy 116. Research indicates that fructose may exert inhibitory effects on tumor growth under specific conditions. Dewdney *et al.* demonstrated that fructose significantly inhibited the growth of HCC by altering the metabolic pathways of cancer cells, and this effect was enhanced when fructose was used in conjunction with the drugs NCT-503 and Physcion 117. This finding highlights the potential positive role of fructose in combination drug therapy for cancer. Additionally, research by Elsaid *et al.* found that substituting glucose with fructose in culture conditions enhanced the expression of Hif1a, affecting stem cell proliferation and cytokine production without inducing stem cell differentiation, suggesting a potential inhibitory effect of fructose on stem cell growth 118. Although fructose may provoke adverse immune reactions in preclinical disease models 119, the fructose content in natural foods typically does not lead to obesity or other adverse effects, provided that excessive intake from processed foods is avoided 120. Furthermore, fructose supplementation can enhance the immunoprotective effects of live vaccines under certain conditions 121 Overall, Fructose plays a multifaceted role in cancer development, contributing to both the promotion of tumor growth and metastasis in some cancers and the inhibition of tumor progression under certain conditions. Understanding this dual effect is critical for the development of targeted therapeutic strategies. **Table 1** summarizes the key mechanisms by which fructose metabolism influences tumorigenesis and metastasis, along with potential therapeutic approaches that target fructose-related pathways.

### 3. Fructose Transporters and Their Role in Cancer

Fructose transporters play a significant role in cancer metabolism by facilitating the uptake of fructose into cancer cells, thereby influencing tumor growth and progression. Among these transporters, GLUT5 has been identified as the primary transporter with a strong affinity for fructose, playing a central role in fructose metabolism in tumor cells. Unlike other glucose transporters, GLUT5 specifically mediates fructose uptake, which has been implicated in multiple cancer types due to its contribution to metabolic reprogramming and tumor progression.

### 3.1. Expression and Function of GLUT5

Multiple studies indicate that GLUT5, together with KHK, plays a crucial role in fructose metabolism; importantly, altered GLUT5 activity disrupts cellular carbohydrate metabolism and thereby elevates cancer risk, directly contributing to carcinogenesis 122. After fructose is transported into cells via GLUT5, it is converted by KHK into fructose-1-phosphate (F1P). Accumulated F1P allosterically inhibits pyruvate kinase M2 (PKM2) activity, impairing energy metabolism in tumor cells, particularly under hypoxic conditions. A small-molecule activator of PKM2 has been shown to suppress prostate tumor progression, highlighting the potential therapeutic strategies targeting GLUT5-mediated metabolic pathways 123. Altered expression and/or activity of GLUT5 has been linked to the progression of various cancers, including lung adenocarcinoma, multiple myeloma, breast cancer, and gliomas 14. For instance, Liang *et al.* found that GLUT5 drives tumor progression by promoting fructose metabolism in tumor cells through metabolic reprogramming, even independent of KHK 124. Similarly, Su *et al.* demonstrated in gliomas that elevated GLUT5 accelerates tumor progression by enhancing fructose uptake, while GLUT5 knockdown markedly suppresses tumor growth 125. Consistent with these findings, Suades *et al.* reported that abnormal GLUT5 activity, influenced by membrane fluidity, leads to metabolic dysregulation, thus promoting tumor progression 126. Additionally, Suwannakul *et al.* reported that high expression of GLUT5 in cholangiocarcinoma cells enhanced both fructose utilization and metabolic adaptation, and silencing GLUT5 significantly inhibited tumor cell proliferation, offering a new therapeutic strategy targeting GLUT5-mediated metabolic reprogramming in cholangiocarcinoma 127. Chen *et al.* found that GLUT5-mediated fructose utilization promotes proliferation of lung cancer cells by accelerating fatty acid synthesis (especially palmitoleic acid), subsequently activating the mTORC1 signaling pathway through inhibition of AMPK activity 34. Huang *et al.* showed that activation of the IL-6/STAT3 pathway increased GLUT5 expression, crucial for the growth of tumor cells, suggesting that targeting GLUT5 might slow tumor development 128. Jin *et al.* emphasized the critical role of GLUT5 in renal clear cell carcinoma, where modulating GLUT5 expression directly controls fructose metabolism in tumor cells. Reducing GLUT5 activity directly decreases fructose metabolism, resulting in suppression of malignant features of tumor cells 129. Research indicates that the BTBD7-SLC2A5 (GLUT5) gene fusion may promote prostate cancer progression by altering glucose metabolism 130. Yang *et al.* discovered that GLUT5-mediated fructose utilization enhanced the migratory capacity of lung cancer cells, an effect achieved by increasing lactate production and AKT phosphorylation during glycolysis 131. Furthermore, Weng *et al.* demonstrated that overexpression of SLC2A5 is associated with poor prognosis in lung adenocarcinoma, and inhibition of GLUT5 could increase the sensitivity of lung adenocarcinoma cells to the chemotherapeutic drug paclitaxel 33. In CRC, S100P promotes metastasis by decreasing GLUT5 promoter methylation and activating transcription; elevated GLUT5 expression is strongly linked to enhanced invasiveness and metastatic potential, underscoring its crucial role in cancer metabolism 132. In breast cancer cells, high GLUT5 expression enables efficient fructose utilization, making fructose a key energy source for survival and thus facilitating cancer progression 66. Włodarczyk *et al.* demonstrated that the GLUT5 inhibitor MSNBA effectively inhibits colon cancer cell proliferation with negligible effects on normal cells, reinforcing that GLUT5-mediated fructose utilization preferentially supports cancer cell proliferation 133. Cairns *et al.* found that AML cells under low glucose conditions markedly upregulate GLUT5 expression, enabling fructose uptake and its subsequent conversion into glycolytic intermediates, thereby sustaining tumor cell survival and proliferation 105. Zhao *et al.* showed that increased SLC2A5 (GLUT5) expression in pediatric Ph+ALL correlates with disease relapse and treatment resistance, and notably, tyrosine kinase inhibitors reduce GLUT5 expression, thereby decreasing fructose uptake 134. Zakłos-Szyda *et al.* found that certain phenolic plant extracts downregulate GLUT5 levels, leading to reduced fructose uptake in Caco-2 cells, thus restricting energy supply to cancer cells and potentially slowing tumor growth 135. Park *et al.* demonstrated that AKT1/3 activation induces GLUT5 expression via downregulation of miR-125b-5p; elevated GLUT5 expression subsequently promotes CRC cell migration and chemotherapy resistance, whereas GLUT5 inhibition restores chemotherapy sensitivity 136. Shen *et al.* demonstrated that GLUT5, in collaboration with KHK, promotes proliferation and chemotherapy resistance in CRC by facilitating fructose metabolism; accordingly, restricting fructose availability or inhibiting this metabolic pathway significantly suppresses tumor growth and enhances chemotherapy sensitivity 32. In clear cell renal carcinoma (cRCC), elevated GLUT5 expression significantly correlates with lower tumor differentiation, increased pelvic invasion, and capsule breaches, indicating a clear link between GLUT5 upregulation and advanced tumor characteristics 137. Groenendyk *et al.* discovered that genetic inactivation of SLC2A5 (GLUT5) significantly inhibits cancer cell migration by modulating mitochondrial function, indicating that GLUT5 expression directly contributes to tumor metastasis risk and thus represents a promising therapeutic target 138. Soueidan *et al.* revealed that two fluorinated fructose derivatives (3-FDF and 1-FDAM) are efficiently transported into breast cancer cells via GLUT5, emphasizing GLUT5's central role in fructose-dependent tumor metabolism and potential cancer progression 139. Hsu *et al.* discovered that GLUT5 is involved in tumor growth via modulation of glucose metabolism in breast cancer cells, and that GLUT5-targeted BSA nanoparticles significantly enhance drug delivery and anti-tumor efficacy 140. Kannan *et al.* demonstrated that breast cancer cells exhibit significantly higher fructose uptake than normal cells when analyzed using GLUT5-specific fluorescent probes, indicating that elevated GLUT5 expression provides cancer cells with a metabolic advantage, thus facilitating tumor growth and metastasis 141. Chałaśkiewicz *et al.* reported that the histone deacetylase inhibitor Trichostatin A upregulates SNAI1 and SNAI2 expression, resulting in suppression of SLC2A5 (GLUT5) expression in colon cancer cells and consequently enhancing their sensitivity to chemotherapeutic agents cisplatin and oxaliplatin 142. Pu *et al.* developed a dual-targeted liposome specifically targeting GLUT5 and integrin αvβ3, demonstrating enhanced drug uptake and accumulation within tumor sites in triple-negative breast cancer; these results emphasize GLUT5 as a promising therapeutic target capable of improving treatment efficacy 143. Reinicke *et al.* observed that the expression patterns of GLUT1 and GLUT5 differ in prostate cancer; specifically, GLUT1 expression is reduced in cancer tissues, whereas GLUT5 expression persists in high-grade prostatic intraepithelial neoplasia, implying its potential involvement in early carcinogenic events 144. Hamann *et al.* discovered that GLUT5 expression is induced by hypoxic conditions in breast cancer, potentially enhancing tumor growth through increased reliance on fructose metabolism under metabolic stress 145. Research has shown that elevated GLUT5 expression in ovarian cancer cells promotes fructose metabolism, enhancing tumor cell growth and migration; correspondingly, silencing GLUT5 or reducing fructose intake significantly suppresses tumor growth and migration 146. Fransson *et al.* found that SLC2A5 (GLUT5) expression is significantly elevated in Stage 4 neuroblastoma, suggesting its potential role in tumor progression 147. To further illustrate the contribution of GLUT5 in tumor progression, its expression, associated mechanisms, and impact across various cancer types are summarized **(Table 2)**.

## 4. Key Enzymes in Fructose Metabolism and Their Impact on Cancer Progression

The enzymatic phosphorylation of fructose and its metabolic derivatives plays a crucial role in intracellular fructose metabolism, a process primarily regulated by various enzymes, particularly through phosphorylation reactions. This metabolic pathway contributes not only to energy production but also plays a significant role in cancer development. Two key enzymes are involved in the phosphorylation of fructose and its derivatives: KHK and hexokinase 2 (HK2) 148.

### 4.1. The Role of KHK in Tumor Development

Fructose metabolism is increasingly recognized as a critical pathway in cancer metabolism, with KHK acting as a central enzyme in these processes. Notably, stressful tumor microenvironmental conditions (such as hypoxia or glucose deprivation, as well as systemic hyperglycemia) induce the expression of polyol pathway enzymes and KHK, thereby enhancing fructose production and its conversion to fructose-1-phosphate (F1P) to sustain tumor growth 145, 149. Kang *et al.* found that under hyperglycemic conditions, this metabolic shift promotes gastric cancer metastasis by activating the KHK-A pathway, which in turn suppresses CDH1 gene expression and facilitates EMT and tumor cell migration. Further studies have shown that targeting KHK-A can effectively inhibit fructose-induced gastric cancer metastasis under these high-glucose conditions 149. Xu *et al.* that KHK-A enhances the progression of HCC by phosphorylating p62 under oxidative stress conditions, thereby activating the Nrf2 signaling pathway, which helps tumor cells adapt to metabolic stress 150. In gastric cancer cells, increased KHK-A promotes cell proliferation, achieved by reducing β-catenin levels. Inhibiting KHK-A significantly slows the proliferation rate of gastric cancer cells, revealing its pro-carcinogenic role and potential therapeutic value 151. Gao *et al.* demonstrated that high expression of KHK in glioma tissues is closely related to tumor malignancy and patient survival rates. Silencing KHK in a fructose-rich tumor microenvironment inhibited the proliferation and migration of glioma cells, suggesting that high fructose intake might promote the progression of gliomas through KHK 152. Similarly, in breast cancer, KHK-A enters the nucleus under fructose stimulation and promotes the aggregation of SLUG at the CDH1 promoter by phosphorylating YWHAH at the Ser25 site, thereby mediating fructose-induced migration 38.

In non-small cell lung cancer (NSCLC), USP36 enhances KHK-A expression through the c-MYC-hnRNPH1/H2 axis, thus promoting tumor growth primarily by boosting glycolysis 153. Chen *et al.* found that NAT10 upregulates FOXP1, which subsequently increases KHK expression, contributing to glycolytic metabolism and promoting both immune suppression and tumor progression in cervical cancer 154. Further research has shown that the expression of KHK-A and ACSS2 pS659 is significantly higher in NSCLC patients than in non-tumorous tissues and is inversely related to patient survival, confirming their role as markers of metabolic reprogramming and independent prognostic indicators for tumor progression 155. KHK-A enhances the proliferation of esophageal squamous cell carcinoma by upregulating PRPS1, potentially serving as a future therapeutic target 156. Additionally, KHK-A promotes liver metastasis of CRC by phosphorylating PKM2. By inhibiting PKM2 activity, KHK-A enhances tumor cell migration and anti-apoptotic capabilities, indicating its pro-carcinogenic role in tumor progression 111. Lin *et al.* found that splicing variations of KHK are associated with the survival and recurrence of HCC patients and are linked to mutations in TP53 and ARID1A, suggesting that these alterations in KHK may promote tumor progression by regulating key signaling pathways 157. Li *et al.* further revealed that KHK-A reduces fructose metabolism through alternative splicing, while phosphorylating PRPS1 to enhance nucleotide synthesis via the pentose phosphate pathway, thereby promoting HCC progression. The activity of KHK-A is correlated with poor prognosis in HCC patients 158. Moreover, Xu *et al.* demonstrated that L-sorbitol interferes with glycolysis through KHK-mediated phosphorylation, leading to increased oxidative stress and mitochondrial damage in tumor cells, which weakens KHK-A-related antioxidant genes and induces tumor cell apoptosis, suggesting that KHK may have a tumor-suppressive role under specific metabolic conditions 159. This finding offers new insights into the dual role of KHK under different metabolic environments. Research by Guccini *et al.* showed that genetic deletion of the metabolic enzyme KHK-C, by inhibiting the KRAS-MAPK and mTORC signaling pathways, suppresses the development of pancreatic cancer, suggesting that KHK-C typically promotes the survival and proliferation of pancreatic cancer cells 160. Lanaspa *et al.* demonstrated that KHK-C promotes fructose metabolism, leading to energy imbalance and increased oxidative stress, which in turn drives tumor cell proliferation and survival. This enzyme activates the mTOR signaling pathway, further facilitating tumor metabolic reprogramming. Inhibition of KHK-C may represent a potential anti-tumor strategy 72. Gutierrez *et al.* reported that fructose metabolism via KHK significantly affects metabolic health, influencing insulin resistance and fatty liver conditions. In animal models, inhibiting KHK with PF-06835919 effectively improved these conditions, supporting KHK as a critical metabolic target 161. Patel *et al.* revealed that KHK deficiency prevents fructose-induced hyperglycemia but causes hyperfructosemia, highlighting KHK's central role in fructose metabolism 162. In HCC, reduced expression of KHK impairs fructose metabolic functions, detectable by hyperpolarized magnetic resonance spectroscopy in vivo, potentially offering new biomarkers for cancer diagnosis and monitoring 39. Futatsugi *et al.* found that the KHK inhibitor PF-06835919 shows potential in inhibiting fructose metabolism, providing a new strategy for treating related metabolic disorders 163.

### 4.2. HK2 in Cancer Metabolism

HK2 plays a pivotal role in cancer metabolism, particularly by driving metabolic reprogramming within the tumor microenvironment, thereby enhancing its oncogenic potential. HK2 catalyzes the phosphorylation of glucose to form glucose-6-phosphate, and to a lesser extent, it also phosphorylates fructose to generate fructose-6-phosphate. In HCC, HK2 promotes the survival and proliferation of cancer stem cells by activating ACSL4 and enhancing fatty acid β-oxidation. This enzyme further supports the energy demands of liver cancer cells and enhances their invasiveness by facilitating the accumulation of acetyl-CoA 164. Chen *et al.* demonstrated that HK2 accelerates glycolysis in HBx-induced HCC via the NF-κB p65 signaling pathway, while further supporting tumor growth through the activation of the PI3K/Akt pathway 165. Additionally, DeWaal *et al.* showed that silencing HK2 inhibited glycolysis and restored oxidative phosphorylation, leading to cell death, which underscores HK2's role in maintaining the glycolytic phenotype of cancer cells. Furthermore, when HK2 was silenced in combination with metformin, tumor cell growth was significantly suppressed, and mTORC1 inhibition occurred via an AMPK-independent mechanism 166. Under hypoxic conditions, the interaction between HK2 and TIGAR amplifies HK2 activity, helping regulate mitochondrial ROS levels—further reinforcing HK2's involvement in the progression of cancer 167. In breast cancer, Zhang *et al.* revealed that HK2 enhances glycolysis through the ROS/PI3K/AKT pathway, and inhibiting HK2 lowered ROS levels, improving the efficacy of cancer treatments 168. In cervical cancer, Wang *et al.* found that HK2 promotes tumorigenesis by enhancing glycolysis, with its stability regulated by m6A methylation and YTHDF1 169. Liu *et al.* further demonstrated that E6E7 promotes cervical cancer progression by releasing the inhibition on HK2, leading to an upregulation of its expression and enhanced glycolysis 170. Wang *et al.* also found that HK2 maintains cancer stemness and promotes tumor growth in small cell lung cancer by enhancing the stability of CD133 171. Moreover, Zhang *et al.* reported that HK2 supports tumorigenesis by facilitating glycolysis in cancer cells, while STING inhibits aerobic glycolysis by targeting HK2, thereby enhancing the antitumor immune response 172. Cao *et al.* further revealed that in breast cancer, HK2 exerts pro-oncogenic effects by promoting glycolysis, with its expression regulated by the circRNF20/miR-487a/HIF-1α axis. HIF-1α, stabilized under hypoxic conditions, further enhances HK2 transcription, linking tumor hypoxia in the TME to increased HK2 expression 173. HK2's significance extends across various cancer types. For instance, in gastric cancer, HK2-driven glycolysis is regulated by circadian rhythms, promoting tumor growth and contributing to Trastuzumab resistance. Notably, silencing PER1, which disrupts HK2's circadian regulation, can reverse this resistance 174. Furthermore, HK2 activity can be inhibited by exogenous substances. Nakayama *et al.* reported that cinnamon bark extract (CBE) inhibits HK2, thereby blocking the production of glucose-6-phosphate and subsequently suppressing cancer cell invasion and migration 175. Recent studies have further demonstrated HK2's clear oncogenic role in glucose metabolism. Zhang *et al.* found that resistant starch effectively suppresses HFCS-induced colon carcinogenesis by downregulating HK2 expression 176. Collectively, these findings underscore HK2's critical function in cancer metabolism, not only by fulfilling the energy needs of cancer cells but also by promoting cancer progression through various mechanisms, including metabolic reprogramming of the TME, immune evasion, and hypoxia adaptation, making it a crucial therapeutic target in cancer treatment. An overview of the critical enzymes in fructose metabolism, their oncogenic functions, and the relevant molecular pathways has been compiled **(Table 3)**.

## 5. Association Between Aldehyde Dehydrogenase Family Gene Expression and Clinical Outcomes in Cancer

The TME is profoundly influenced by metabolic enzymes such as those in the aldehyde dehydrogenase (ALDH) family. The ALDH family, which includes isoforms ALDH A, B, and C, is expressed in specific tissues and catalyzes the conversion of fructose-1,6-bisphosphate into glyceraldehyde-3-phosphate and dihydroxyacetone phosphate. This metabolic catalytic activity is crucial for fueling the proliferation of cancer cells 177. Consistent with this, studies have shown that overexpression of ALDH family members is closely associated with tumor formation and can promote cancer progression by influencing various phenotypes of cancer cells 178. Moreover, ALDH expression serves as an independent prognostic factor in cancer patients, underscoring its significance in tumor biology and its interplay with the TME.

### 5.1. ALDOB: A Critical Player in Cancer Metabolism and Progression

ALDOB, also referred to as liver aldolase, is predominantly located in the liver and kidneys 179. Notably, aberrant ALDOB expression is associated not only with hereditary fructose intolerance (HFI), liver cirrhosis, and hepatitis, but also with oncogenesis 180, 181. Specifically, the abnormal activity of ALDOB in CRC is closely associated with pathological processes, as has been evidenced by numerous studies 37, 182-185. Additionally, ALDOB exhibits altered expression in HCC, where its dysregulated activity is similarly tied to the progression and metastasis of cancer 186-188. Collectively, these findings suggest that ALDOB plays a crucial role across multiple cancer types, potentially through metabolic reprogramming within the tumor microenvironment.

High expression of ALDOB in rectal cancer significantly correlates with poor patient prognosis, manifesting as reduced survival rates and weaker responses to chemotherapy and radiation therapy. Elevated levels of ALDOB are associated with aggressive tumor characteristics, such as lymphovascular invasion and perineural invasion, highlighting its oncogenic role in rectal cancer 185. Similarly, in CRC, elevated ALDOB levels are associated with lower overall survival, and ALDOB inhibition can suppress the EMT of tumor cells, suggesting that ALDOB could be a potential therapeutic target 182. Research by Bu *et al.* demonstrated that in CRC liver metastasis, ALDOB enhances tumor cell growth by facilitating fructose metabolism, allowing tumor cells to adapt their metabolic pathways to utilize available sugar sources in the liver's new microenvironment through the action of ALDOB. Furthermore, therapies targeting ALDOB have proven effective in inhibiting tumor growth in the liver, indicating that modulating this metabolic pathway is an effective strategy for controlling tumor metastasis 37. Lin *et al.* found that ALDOB is upregulated in colorectal adenomas, shifting energy metabolism from oxidative phosphorylation to glycolysis, which is closely associated with tumor growth and progression. Moreover, ALDOB synergizes with SLC16A4 to drive both the glycolytic and fructose metabolic pathways, further enhancing tumor cell survival and proliferation 189. ALDOB also contributes to a pro-tumor metabolic microenvironment through enhanced lactate production and adaptation to hypoxia. Chu *et al.* reported that ALDOB overexpression in CRC cells increases lactate production and secretion (via upregulating LDHB), which sustains cancer cell proliferation and confers resistance to chemotherapy; they also identified CEACAM6 as a downstream effector mediating ALDOB's impact on cellular metabolism 190. In parallel, Huang *et al.* discovered that in a rigid tumor microenvironment, ALDOB can reverse the metabolic suppression of CRC cells, enhancing glycolysis while reducing oxidative phosphorylation, thus adapting to hypoxic conditions and potentially promoting tumor growth 183. Civit *et al.* demonstrated that ALDOB deficiency leads to disrupted fructose metabolism, activating the mTOR signaling pathway and increasing tumor cell proliferation. Moreover, abnormal fructose metabolism accelerates glycolysis, further promoting tumor progression 191.

While the mechanisms by which ALDOB promotes tumor growth have been extensively discussed, research has also uncovered its potential role in cancer suppression. In HCC, for example, low ALDOB expression is associated with more aggressive disease and poorer prognosis, whereas restoring ALDOB expression inhibits tumor invasiveness in part by upregulating the DNA demethylase TET1 186. Likewise, in gastric cancer, loss of ALDOB correlates with adverse outcomes, while ALDOB re-expression curbs tumor cell growth and migration by suppressing the AKT signaling pathway 192. Consistent with this, ALDOB reintroduction in gastric cancer cells was shown to downregulate key glycolytic enzymes, leading to reduced lactate production and an overall decrease in cancer cell energy metabolism; consequently, these metabolic changes increased the cells' sensitivity to the chemotherapeutic agent Talazoparib 193. In ccRCC, ALDOB has been identified as a metabolic tumor suppressor. Tan *et al.* reported that ALDOB forms a complex with acetoacetyl-CoA reductase to produce metabolites that inhibit the oncogenic co-repressor CtBP2, thereby reactivating silenced anti-tumor genes and restraining tumor cell proliferation and migration 194. Furthermore, ALDOB downregulation in ccRCC leads to accumulation of fructose-1,6-bisphosphate and disrupts redox homeostasis, whereas ALDOB upregulation restores metabolic balance and inhibits tumor progression 195. In line with this, loss of the ALDOB gene (e.g., via copy number reduction) drives hyperactive glucose metabolism in metastatic ccRCC, promoting tumor progression and correlating with poor patient prognosis 196. Moreover, the anti-cancer effects of ALDOB in other cancer types have been confirmed. Wang *et al.* noted that ALDOB, by interacting with DUSP4, regulates glucose metabolism and ROS metabolism in HER2-positive breast cancer, thereby enhancing treatment sensitivity 197. Liu *et al.* found that the absence of ALDOB, by activating the PI3K-AKT signaling and promoting lipogenesis, drives the progression of HCC, whereas its interaction with the insulin receptor regulates glycolipid metabolism and the restoration of ALDOB expression significantly inhibits tumor formation, demonstrating its potential as an anticancer agent 198. Qin *et al.*'s research further supports the tumor-suppressive role of ALDOB, showing that a ketogenic diet inhibits ALDOB enzymatic activity through lysine β-hydroxybutyrylation, leading to metabolic alterations that suppress glucose metabolism and mTOR signaling, ultimately inhibiting HCC cell proliferation 199. Further research has shown that ALDOB inhibits the progression of liver cancer by reducing the activity of G6PD and thereby decreasing the functionality of the pentose phosphate pathway, closely correlating with patient prognosis 200. Additionally, another study revealed that in pancreatic ductal adenocarcinoma (PDAC), the GLUT1/ALDOB/G6PD axis modulates glycolysis and pyrimidine biosynthesis, which may contribute to metabolic conditions affecting chemotherapy sensitivity. Particularly in glucomet-PDAC, high GLUT1 and low ALDOB expression correlate with chemotherapy resistance. Inhibiting this metabolic axis enhances responsiveness to chemotherapy, offering new treatment opportunities for patients resistant to chemotherapy 201. Furthermore, He *et al.* reported that ALDOB, by forming a complex with Akt and PP2A, directly inhibits Akt activity, thereby suppressing the occurrence of liver cancer. The absence or disruption of this interaction enhances tumor progression, while inhibition of Akt or activation of PP2A demonstrates anti-tumor effects 188. Yin *et al.* found that in liver cancer, downregulation of ALDOB, by interacting with KAT2A, suppresses TGF-β expression, which in turn impairs the functionality of CD8+ T cells, promoting tumor immune escape and progression of HCC 202. Wang *et al.* further demonstrated that ALDOB promotes tumor progression by inhibiting glycolysis in CD8+ T cells, thereby weakening the antitumor function of CD8+ T cells. CBX4 upregulates ALDOB expression and reduces Akt phosphorylation, leading to immunosuppression 203. Collectively, these findings illustrate that ALDOB can reshape the immune microenvironment of tumors - through TGF-β-mediated expansion of regulatory T cells and direct suppression of cytotoxic T cell metabolism - to facilitate immune evasion. In pancreatic cancer, Xu *et al.* discovered that downregulation of ALDOB, by reprogramming glucose metabolism, enhances the malignant behavior of tumor cells and leads to resistance to postoperative adjuvant transarterial chemoembolization (PA-TACE) 204. Further research indicates that ALDOB, by inhibiting DNA repair and inducing cell apoptosis, exhibits anticancer effects in CRC. Lian *et al.*'s study suggests that this mechanism could significantly improve patient prognosis 184. In studies on liver cancer, the significant reduction in ALDOB expression suggests its potential role in suppressing cancer development 187. Zheng *et al.* found that MRTO4, by inhibiting ALDOB activity, enhances glycolysis in HCC cells, thereby promoting tumor growth and spread. Since ALDOB typically suppresses glycolysis to exert its anticancer effects, its reduced activity in HCC could be a critical factor in tumor progression, supporting the therapeutic potential of activating ALDOB by inhibiting MRTO4 205. In conclusion, ALDOB's function in cancer encompasses both promotion of tumor growth and potential inhibition, revealing its complex involvement in oncogenesis and underscoring its importance as a therapeutic target.

### 5.2. ALDOA: Enhancer of Glycolysis and Tumor Aggressiveness

ALDOA's expression in various cancers is closely linked to tumor invasiveness and metastatic potential. Under tumor microenvironmental constraints such as hypoxia and nutrient fluctuations, ALDOA is often upregulated to sustain glycolytic flux and support cancer cell survival 206, 207. Chang *et al.* found that in lung cancer, high expression of ALDOA enhanced lactate production, subsequently inhibiting PHD activity and stabilizing HIF-1α, which ultimately activated MMP9 and promoted tumor invasiveness and metastatic capabilities. This lactate-mediated signaling represents a critical mechanism by which ALDOA influences the tumor microenvironment, underscoring the significance of ALDOA in influencing lung cancer progression via metabolic pathways 206. Additionally, Wang *et al.* demonstrated that ALDOA accelerated glycolysis in receptor cells of exosomes from irradiated lung cancer cells, enhancing their migration and invasion, illustrating ALDOA's role in regulating glycolytic pathways to promote tumor cell aggressiveness 207. In liver and bile duct cancers, ALDOA's function is equally crucial. Li *et al.* reported that in liver cancer, ALDOA facilitated cancer progression through glycolysis, with its activation aiding cancer cell survival and proliferation, noting that inhibiting ALDOA's activity might slow tumor growth 164. Fan *et al.* discovered that lactylation of ALDOA in hepatic carcinoma stem cells enhanced their stemness and glycolytic activity via interactions with DDX17, contributing to the specialized metabolic properties of the cancer stem cell niche 208. Grandjean *et al.* revealed a novel positive feedback mechanism between glycolysis and HIF-1α signaling, in which ALDOA indirectly enhances HIF-1α transcriptional activity in hypoxic tumor environments by promoting glycolysis, which is essential for adaptation to hypoxic conditions in the tumor microenvironment, thus stimulating tumor growth. Targeting ALDOA in this context has been shown to significantly improve survival rates in breast cancer models 209. Xu *et al.* demonstrated that ALDOA promotes the proliferation and survival of SSCs by regulating glycolysis and inhibits apoptosis through its interaction with LncRNA ACVR2B-as1, thereby promoting cancer cell growth 210. Yu *et al.* further demonstrated that the alternative splicing of ALDOA, through the insertion of exon 7.2, activates the mTOR signaling pathway, leading to TAM resistance in breast cancer. An ALDOA inhibitor effectively suppressed the resistant tumor cells 211. In the development of HCC, Li *et al.* confirmed that ALDOA acts as an oncogene, with its overexpression positively correlated with the malignancy of tumors. ALDOA enhances the glycolytic capacity and invasiveness of cancer cells, thereby promoting tumor progression 212. Han *et al.* further discovered that in PDAC, ALDOA maintains its stability through LIPH activation, promoting glycolysis and facilitating tumor progression. The stability of ALDOA is crucial for tumor cell proliferation and metabolism, and inhibiting ALDOA can reduce tumor growth 213. Wang *et al.* discovered that ALDOA promotes tumor glucose metabolism and growth, and that HDPS-4II, by specifically inhibiting ALDOA, significantly suppresses glucose metabolism and tumor growth in HCC, highlighting its potential as a novel therapeutic approach for HCC 214. In bile duct cancer, high ALDOA expression not only increased tumor cell proliferation and invasion but also reduced sensitivity to treatment, thus being identified as a pro-oncogenic factor 215. Wang *et al.* found that in retinoblastoma, ALDOA promotes tumor cell proliferation by enhancing glycolysis. The ALDOA inhibitor, itaconate, has been shown to inhibit tumor growth, indicating the crucial role of ALDOA in tumor energy metabolism 216. In CRC, ALDOA acts as a critical regulator of fructose metabolism, facilitating tumor progression through enhanced glycolysis and cellular proliferation, with its high expression linked to therapy resistance and poor prognosis 217. Moreover, Zhou *et al.* identified a pivotal role of ALDOA in cervical cancer radioresistance, where ALDOA regulates glycolysis and DNA damage response, contributing to tumor cell resistance and growth. Downregulation of ALDOA has been found to enhance the efficacy of radiotherapy 218. Ji *et al.* revealed that ALDOA boosted pancreatic cancer's aggressiveness and metastatic potential by enhancing glycolysis and activating oxidative stress responses, impacting key factors such as c-Myc, HIF1α, and NRF2, thereby promoting tumor growth and spread 219. Cui *et al.* demonstrated that ALDOA inhibits the progression of PDAC through its derived P04 peptide, with the mechanism partially involving the inhibition of pro-cancer signaling pathways related to glycolysis 220. Sobanski *et al.* confirmed that ALDOA, by participating in fructose metabolism and DNA repair, supports tumor cell survival and proliferation, positioning it as a potential therapeutic target in cancer metabolism and DNA repair strategies 221. In conclusion, a comprehensive analysis indicates that ALDOA significantly affects cancer aggressiveness and metastatic potential through its diverse metabolic functions and profound effects on the tumor microenvironment, highlighting its importance as a potential therapeutic target in addressing hypoxia, acidification, and nutrient stress.

### 5.3. ALDOC: A Double-Edged Sword in Cancer Development

ALDOC exhibits a complex duality in its role across various cancer types. Its impact is highly context-dependent, often dictated by the tumor microenvironment: certain conditions enable ALDOC to support tumor glycolysis and growth, whereas in other contexts ALDOC activity suppresses tumor progression. Fan *et al.* found that the interaction between ALDOC and the C-terminal of MUC16 enhanced the growth of gallbladder cancer, primarily by disrupting glucose sensing and activating associated metabolic pathways, thereby reshaping the tumor microenvironment and further reinforcing ALDOC's pro-carcinogenic role in glycolysis 222. Furthermore, Reinsborough *et al.* reported that BCDIN3D upregulates ALDOC expression via let-7 microRNA, thereby promoting glycolysis in breast cancer cells, with high ALDOC expression closely linked to poor prognosis in breast cancer 223. In 3D tumor models, De Vitis *et al.* further validated the pro-carcinogenic role of ALDOC in glycolysis, noting its support for tumor growth through enhanced glycolysis and lactate production 224. Similarly, Shang *et al.* found that ALDOC promotes the development of NSCLC by influencing MYC-mediated transcription of UBE2N and the Wnt/β-catenin signaling pathway, which is closely associated with increased tumor aggressiveness and poor prognosis 225. Kathagen-Buhmann *et al.* demonstrated that ALDOC is upregulated in GBM cells, where it promotes the glycolytic pathway and enhances cell migration capacity 226. However, ALDOC does not universally promote tumor development. Chang *et al.* demonstrated that the activation of the ALDOC-NR2F1 axis by PPAR-γ agonists not only enhances the chemotherapeutic efficacy in GBM but also exhibits ALDOC's tumor-suppressive functions by modulating glycolysis 227. Simultaneously, in oral squamous cell carcinoma, ALDOC inhibits glucose metabolism, reducing ATP and lactate production, thereby mitigating tumor migration and invasion 228. Lao *et al.* found that, in HCC, ALDOC suppresses glycolysis and the pentose phosphate pathway via GCDH-mediated crotonylation, thereby fostering an anti-tumor microenvironment characterized by reduced lactate levels and limited immunosuppressive conditions, thus exerting its tumor-suppressive role 229. ALDOC's involvement in cancer is paradoxical, contributing to both tumor proliferation and suppression, underscoring how shifts in the tumor microenvironment (e.g., nutrient availability, hypoxia, or lactate accumulation) can toggle ALDOC's function between pro-tumor and anti-tumor states.

A detailed overview of the roles of ALDOB, ALDOA, and ALDOC in cancer metabolism, progression, and therapy response, including their dual functions in both promoting and inhibiting tumor growth, is presented in **Table 4**.

## 6. Conclusion and Future Perspectives

**Figure 2:**
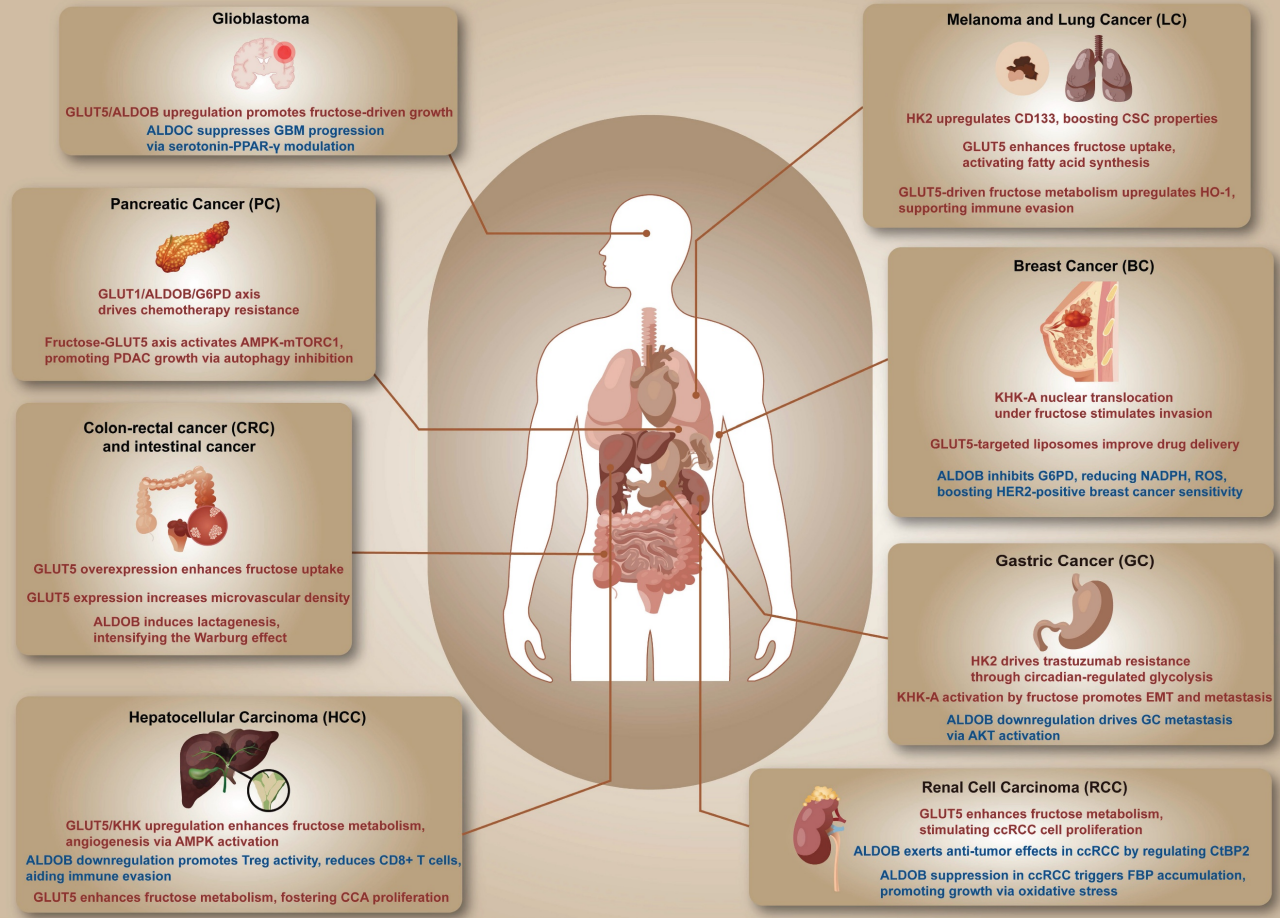
** Fructose metabolism in cancer progression.** Illustration of the involvement of fructose metabolism-related enzymes and transporters in various human cancer types. The figure shows tumor-associated expression and functional roles of key molecules, including GLUT5, KHK, and ALDOB. Tumor-promoting activities are labeled in red; tumor-suppressive or regulatory roles are labeled in blue.

Fructose metabolism is critical for maintaining the metabolic adaptability of cancer cells, particularly under nutrient-limited conditions. Key enzymes such as KHK and ALDOB, along with transporters like GLUT5, enable fructose to be utilized as an alternative carbon source. This metabolic shift supports essential processes including glycolysis, lipid biosynthesis, and nucleotide production, all of which contribute to tumor proliferation and metastasis. The role of fructose metabolism in several cancers, including glioblastoma, colorectal, and hepatocellular carcinomas, positions it as a promising therapeutic target. **Figure 2** illustrates the complex connections between fructose metabolism and tumor progression in different cancer types. It highlights the upregulation of enzymes and transporters such as GLUT5, KHK, and ALDOB across multiple malignancies, driving processes like fructose-dependent growth, drug resistance, and immune evasion. This visual representation emphasizes fructose metabolism as a central factor in cancer development and as a potential target for therapy.

Further research into the molecular mechanisms governing fructose metabolism within the tumor microenvironment is crucial. The ability of cancer cells to rely on fructose metabolism, especially in glucose-deprived environments, calls for deeper investigation into its role in promoting oncogenesis and therapy resistance. While enzyme inhibition, particularly of KHK, shows potential, additional studies are required to assess its therapeutic value across various cancers. The broader effects of fructose metabolism, including its impact on immune evasion, angiogenesis, and interactions with key oncogenic pathways such as mTORC and HIF signaling, also warrant exploration. Understanding these pathways could help determine whether fructose metabolism represents a universal vulnerability in cancer or a context-specific phenomenon.

In conclusion, although substantial evidence supports the involvement of fructose metabolism in cancer progression, more research is needed to clarify its role across different cancer types. Future advances in this field may guide the development of targeted therapies, especially for tumors that are resistant to conventional treatments.

## Figures and Tables

**Table 1:** The Role of Fructose Metabolism in Cancer Cell Metabolic Reprogramming.

Cancer Type	Key Fructose-Driven Mechanisms	Impact on Tumorigenesis	Potential Fructose-Related Therapeutic Targets	References
Glioblastoma Multiforme	Fructose facilitates tumor growth via ATF4-activated fructolysis (GLUT5, ALDOB)	Fructose enhances malignancy under glucose deprivation	Fructose metabolism (ATF4, GLUT5, ALDOB)	(88)
Lung Cancer	Fructose modulates adipocyte metabolism to enhance CD8+ T cell antitumor responses via mTORC1 and leptin	Fructose attenuates CD8+ T cell exhaustion, boosting antitumor immunity	Fructose-leptin axis (mTORC1)	(116)
Lung Cancer	Fructose via GLUT5 drives fatty acid synthesis and activates AMPK/mTORC1	Fructose drives tumor growth, supports metabolic flexibility	Targeting GLUT5 to inhibit fructose utilization	(34)
Colorectal Cancer	Fructose upregulates VEGF via ROS, activates Akt/Src, and enhances VEC proliferation, migration, and tube formation	Fructose promotes angiogenesis, driving tumor progression and increasing MVD	Targeting fructose metabolism, Glut5, and ROS-mediated VEGF upregulation	(93)
Colorectal Cancer	Fructose via GLUT5-KHK sustains proliferation and inhibits autophagy	Fructose promotes growth and chemotherapy resistance	Targeting GLUT5 or KHK, reducing fructose intake	(32)
Colon Cancer Liver Metastasis	Fructose via ALDOB/GATA6 stimulates central carbon metabolism	Fructose drives metabolic reprogramming and promotes metastasis	Targeting ALDOB or reducing fructose intake	(37)
Hepatocellular Carcinoma	Fructose induces acetate via microbiota, boosting O-GlcNAcylation	Fructose enhances O-GlcNAcylation, driving tumor growth	Fructose drives O-GlcNAcylation via OGT and GLUL, modulates gut microbiota-derived acetate production	(95)
Hepatocellular Carcinoma	Fructose enhances O-GlcNAcylation via microbiota-derived acetate	Fructose accelerates tumor growth	Glutamate-ammonia ligase, O-linked N-acetylglucosamine transferase (OGT)	(100)
Hepatocellular Carcinoma	Fructose induces histone hyperacetylation, increasing DNA damage	Fructose induces chromatin relaxation and DNA damage, raising HCC risk	Targeting acetyl-CoA production or histone acetylation	(109)
Pancreatic Ductal Adenocarcinoma	Fructose activates AMPK-mTORC1, inhibiting autophagy in glucose deficiency	Fructose enhances metabolic plasticity, survival, and invasion	Targeting GLUT5-mediated fructose metabolism	(97)
Hepatocellular Carcinoma	Fructose activates AMPK signaling and boosts mitochondrial respiration	Fructose promotes angiogenesis, tumor growth, and metastasis	Targeting SLC2A5 or KHK, fructose restriction	(79)
Esophageal Adenocarcinoma	Fructose alters gut microbiota, triggers metabolic changes and inflammation	Fructose triggers early tumorigenesis from Barrett's esophagus	Targeting fructose-induced microbiota and metabolic shifts	(91)
Melanoma	Fructose increases HO-1 expression, promoting cytoprotection	Fructose promotes immune evasion and immunotherapy resistance	Targeting HO-1 to overcome fructose-induced resistance	(103)
Ovarian Cancer	Fructose enhances CMA, upregulates LAMP2A, SOX2, OCT4, reduces sphere formation	Fructose promotes CSC self-renewal, increases stemness, and impacts metastasis	Targeting CMA via LAMP2A or GLUT5, fructose metabolism	(22)
Breast Cancer	Fructose via KHK-A phosphorylates YWHAH, represses CDH1	Fructose promotes metastasis via invasion and migration.	Targeting KHK-A pathway, reducing fructose intake	(38)

**Table 2:** Expression and Function of Fructose Transporters in Cancer.

GLUT5 Expression	Cancer Type	Role in Cancer Progression	Mechanism/Target	References
Upregulated	Cholangiocarcinoma	GLUT5 promotes tumor growth and ATP production via fructose metabolism	Fructose metabolism, Warburg effect, KHK, ALDOB, LDHA, MCT4, HIF1A	(127)
Upregulated	Colorectal Cancer	GLUT5 promotes metastasis	SLC2A5 transcription activated by S100P through promoter demethylation	(132)
Upregulated	Colorectal Cancer	GLUT5 drives tumor growth and chemotherapy resistance	GLUT5-KHK axis facilitates fructose utilization via glycolysis and TCA cycle	(32)
Upregulated	Colon Cancer	GLUT5 sensitizes cells to platinum-based chemotherapy	Trichostatin A inhibits GLUT5 via SNAI1/SNAI2 transcription factors	(142)
Upregulated	Colon Cancer	GLUT5-mediated fructose transport contributes to metabolic disturbances	GLUT5 facilitates fructose uptake, regulated by TXNIP and ChREBP, reduced by phenolic-rich extracts	(135)
Upregulated	Oral Squamous Cell Carcinoma and Prostate Cancer	GLUT5 enhances fructose uptake and tumorigenesis	IL-6/STAT3 activates GLUT5, increases fructolysis	(128)
Upregulated	Lung Cancer	GLUT5 drives lung cancer via fructose utilization	GLUT5 facilitates fructose uptake, activating fatty acid synthesis and AMPK/mTORC1 signaling	(34)
Upregulated	Lung Adenocarcinoma	GLUT5 enhances cell growth and metastasis	GLUT5-mediated fructose metabolism enhances intracellular fatty acid accumulation and ATP production	(33)
Upregulated	Clear Cell Renal Cell Carcinoma	GLUT5 increases proliferation and colony formation	GLUT5-mediated fructose metabolism inhibits apoptosis and supports tumor growth	(129)
Upregulated	Lung Cancer	GLUT5 promotes migration and metastasis	GLUT5 enhances fructose metabolism, activates glycolysis and AKT phosphorylation	(131)
Upregulated	Triple-negative breast cancer	GLUT5 enhances targeted drug delivery and tumor inhibition	Dual-targeting liposomes with GLUT5 and integrin αvβ3 improve paclitaxel delivery	(143)

**Table 3:** Critical Enzymes in Fructose Metabolism and Their Impact on Cancer Progression.

Enzyme	Cancer Type	Oncogenic Role	Molecular Mechanism	References
KHK-A	Gastric Cancer	KHK-A promotes metastasis via EMT	Hyperglycemia induces fructose via polyol pathway, activating KHK-A, repressing CDH1	(149)
KHK-A	Gastric Cancer	KHK-A promotes tumor cell proliferation and mitochondrial function	KHK-A increases β-catenin; inhibition reduces β-catenin, impairs mitochondrial function	(151)
KHK-A	Colorectal Cancer Liver Metastasis	KHK-A promotes fructose-dependent CRLM	KHK-A phosphorylates PKM2, reducing pyruvate kinase activity and promoting nuclear PKM2, driving EMT and glycolysis	(111)
KHK-A	Hepatocellular Carcinoma	KHK-A enhances cell survival under oxidative stress	KHK-A phosphorylates p62, blocks ubiquitination, activates Nrf2, reducing ROS	(150)
KHK-A	Hepatocellular Carcinoma	KHK-A drives tumor progression	KHK-A phosphorylates PRPS1, promoting nucleic acid synthesis	(158)
KHK-C	Pancreatic Ductal Adenocarcinoma	KHK-C accelerates PDAC progression	KHK-C enhances KRAS-MAPK activation and rpS6, promoting cell migration	(160)
KHK-A	Breast Cancer	KHK-A promotes metastasis	KHK-A phosphorylates YWHAH, recruiting SLUG to CDH1 promoter	(38)
KHK-A	Non-Small Cell Lung Cancer	KHK-A enhances tumor growth	USP36 stabilizes c-MYC, upregulating hnRNPH1/H2 and KHK-A	(153)
KHK	Cervical Cancer	KHK enhances tumor glycolysis	Upregulates glucose metabolism pathways	(154)
KHK-A	Various Cancer	KHK-A downregulation by L-sorbose induces apoptosis	KHK-A downregulation impairs glycolysis and mitochondrial function	(159)
KHK-C	Metabolic-related Cancers	Enhances fructose metabolism, leading to metabolic and proliferative effects in tumors	Inhibition of KHK-C reduces ATP depletion and phosphate loss, protecting against metabolic dysfunction	(72)
KHK	N/A	Contributes to metabolic dysfunction	KHK enhances fructose metabolism, promoting DNL and ChREBP activation	(161)
KHK	N/A	Promotes metabolic disorders	KHK converts fructose to F1P, initiating a metabolic cascade	(163)
HK2	Liver Cancer	Enhances cancer stemness	HK2 activates ACSL4 and fatty acid β-oxidation to promote stem cell renewal	(164)
HK2	Hepatocellular Carcinoma	HK2 enhances HBx-initiated carcinogenesis	HK2 activation via NF-κBp65 enhances aerobic glycolysis and PI3K/Akt signaling	(165)
HK2	Hepatocellular Carcinoma	HK2 drives tumor growth and metabolic adaptation	HK2 depletion inhibits glycolysis, raises oxidative phosphorylation, and increases metformin sensitivity	(166)
HK2	N/A	HK2 promotes cell survival under hypoxia	HK2 activity is increased under hypoxia via its complex with TIGAR, reducing ROS and cell death	(167)
HK2	Breast Cancer	Supports aerobic glycolysis and tumor growth	HK2 promotes glycolysis via ROS/PI3K/AKT/HIF-1α pathway. Polydatin and 2-DG suppress HK2, enhancing anti-cancer effects.	(168)
HK2	Cervical Cancer	Promotes the Warburg effect and tumorigenesis	METTL3 enhances HK2 mRNA stability via YTHDF1-mediated m6A modification, driving aerobic glycolysis	(169)
HK2	Cervical Cancer	Promotes tumorigenesis and metastasis	E6E7 upregulates HK2 expression via GSK3β, while FTO inhibits HK2 by retaining its pre-mRNA	(170)
HK2	Small Cell Lung Cancer	Enhances CSC stemness	HK2 stabilizes CD133 by recruiting USP11, preventing polyubiquitination and promoting tumor growth	(171)
HK2	Gastric Cancer	Promotes trastuzumab resistance	HK2 regulates glycolysis through circadian rhythm disruption, enhancing resistance	(174)
HK2	Colorectal Cancer	Promotes aerobic glycolysis and tumor progression	STING inhibits HK2, reducing glycolysis and enhancing antitumour immunity	(172)
HK2	Colorectal Cancer	Promotes glycolysis and tumorigenesis	HK2 promotes HFCS-induced tumorigenesis by enhancing glycolysis. RS downregulates HK2, increases SCFAs, and inhibits tumor growth	(176)

**Table 4:** Summary of ALDH Family Gene Expression and Their Impact on Cancer Progression.

ALDH Family Member	Cancer Type	Oncogenic Role	Molecular Mechanism	References
ALDOB	Colorectal Cancer	ALDOB upregulation enhances CRC glycolysis in hypoxia and stiff substrate	Promotes glucose uptake, aerobic glycolysis, and reduces oxidative phosphorylation	(183)
ALDOB	Colorectal Adenoma/Carcinoma	ALDOB promotes the glycolytic shift	ALDOB shifts metabolism to glycolysis and reduces OxPhos; knockdown restores mitochondrial respiration	(189)
ALDOB	Colorectal Cancer	ALDOB promotes cell proliferation and chemoresistance	ALDOB induces lactagenesis, activates PDK1, and upregulates CEACAM6 through lactate secretion, driving the Warburg effect	(190)
ALDOB	Gastric Cancer	Tumor-suppressive	ALDOB inhibits AKT activation	(192)
ALDOB	Hepatocellular Carcinoma	Tumor-suppressive	ALDOB depletion disrupts its interaction with the IR, triggering PI3K-AKT pathway activation, promoting de novo lipogenesis	(198)
ALDOB	Hepatocellular Carcinoma	Suppression of ALDOB enzymatic activity reduces cancer cell proliferation	Kbhb at ALDOB Lys108, induced by a ketogenic diet, suppresses its enzymatic activity, reducing mTOR signaling and glycolysis	(199)
ALDOB	Hepatocellular Carcinoma	Tumor-suppressive	ALDOB limits G6PD, disrupting HCC metabolism. Boosts p53-G6PD suppression, curbing tumors. ALDOB loss raises G6PD, worsening prognosis	(200)
ALDOB	Hepatocellular Carcinoma	ALDOB deficiency increases TGF-β, enhancing Treg cells and suppressing CD8+ T cell function	ALDOB-KAT2A interaction inhibits H3K9 acetylation, suppressing TGFB1 transcription	(202)
ALDOB	Pancreatic Ductal Adenocarcinoma	ALDOB contributes to chemotherapy resistance in glucomet-PDACs	GLUT1/ALDOB/G6PD axis remodels metabolism in PDAC, increasing glycolytic flux and pyrimidine biosynthesis	(201)
ALDOB	Clear Cell Renal Cell Carcinoma	Tumor-suppressive	ALDOB recruits acireductone dioxygenase 1, inhibiting CtBP2 and reducing ccRCC growth	(194)
ALDOB	Clear Cell Renal Cell Carcinoma	Tumor-suppressive	ALDOB loss results in FBP accumulation, suppressing NOX4 and aiding tumor growth by counteracting oxidative stress	(195)
ALDOB	HER2-positive Breast Cancer	Tumor-suppressive	ALDOB dephosphorylation by DUSP4 suppresses G6PD activity, leading to elevated ROS levels and increased sensitivity to HER2-targeted therapies	(197)
ALDOB	Unspecified	ALDOB inhibits glycolysis in CD8+ T cells, impairing their antitumor activity and facilitating immune escape	ALDOB is upregulated by CBX4, which decreases Akt phosphorylation	(203)
ALDOA	Non-Small Cell Lung Cancer	Promotes metastasis	ALDOA activates HIF-1α stabilization, leading to MMP9 upregulation	(206)
ALDOA	Hepatocellular Carcinoma	Enhances LCSC stemness	Lactylation of ALDOA at K230/322 enhances DDX17 interaction, promoting LCSC stemness	(208)
ALDOA	Hepatocellular Carcinoma	ALDOA enhances aerobic glycolysis, driving HCC progression	HDPS-4II selectively inhibits ALDOA, decreasing glycolysis and AMPKα phosphorylation	(214)
ALDOA	Intrahepatic Cholangiocarcinoma	ALDOA promotes proliferation and migration	ALDOA enhances glycolysis in ICC cells, driving metabolic reprogramming	(215)
ALDOA	Pancreatic Ductal Adenocarcinoma	Promotes glycolysis and tumor growth	LIPH activates LPA/LPAR axis to stabilize ALDOA, enhancing PI3K/AKT/HIF1A signaling, promoting glycolysis	(213)
ALDOA	Breast Cancer	ALDOA contributes to tamoxifen resistance	ALDOA expression increases via exon 7.2 splicing, activating mTOR pathway, promoting drug resistance	(211)
ALDOA	Retinoblastoma	Promotes tumor growth and metabolism	ALDOA overexpression enhances RB cell viability, regulates SUSD2, ARHGAP27, and CLK2; inhibited by itaconate	(216)
ALDOC	Lung & Breast Cancer	Supports anchorage-independent growth of cancer cells	ALDOC promotes glycolysis, elevating glucose/fructose uptake and lactate output, facilitating 3D spheroid growth	(224)
ALDOC	Breast Cancer	ALDOC downregulation decreases glycolysis, linking expression to poor prognosis	ALDOC is regulated by BCDIN3D via let-7 microRNA, affecting F1,6-BP levels	(223)
ALDOC	Glioblastoma	Tumor-suppressive	ALDOC hypermethylation drives GBM invasion by disrupting serotonin and the ALDOC-NR2F1 axis; PPAR-γ agonists restore its function and improve temozolomide sensitivity	(227)
ALDOC	Hepatocellular Carcinoma	Tumor-suppressive	GCDH-induced crotonylation of ALDOC limits glycolysis and PPP, inducing HCC cell senescence and anti-tumor effects	(229)
ALDOC	Gallbladder Carcinoma	Promotes glycolysis and proliferation via MUC16c binding	MUC16c-ALDOC binding disrupts glucose-sensing ability, activating the AMPK pathway	(222)
